# Adoption of strategies to improve decision support in community mental health centers

**DOI:** 10.1186/1748-5908-10-S1-A27

**Published:** 2015-08-20

**Authors:** Shari L Hutchison, Irina Karpov, Pat E Deegan, Kim L MacDonald-Wilson, James M Schuster

**Affiliations:** 1 UPMC Insurance Division, Community Care Behavioral Health Organization, Pittsburgh, PA 15222, USA; 2 Pat Deegan PhD & Associates, LLC, Byfield, MA 01922, USA

## Objective

Assessment of specific skills acquired during training of a new practice is important in understanding adoption, but often difficult to do. This project summarizes information from a clinical skills assessment over one year post training of a program to increase treatment decision support by staff for individuals receiving mental health services.

## Method

Training and implementation on a Decision Support Toolkit (Pat Deegan & Associates) was provided and supported by a managed behavioral health care organization to interested providers. Individuals in service and staff at four community mental health centers completed a skills and practices survey where they rated how often 18 activities occurred during their clinical sessions on a Likert scale. To examine subscales, an exploratory factor analysis was conducted using the Principal Axis Factoring method for non-normally distributed data with Promax rotation to account for correlations among subscales.

## Results

Surveys were completed by 41 staff at baseline, 43 at 6 months, 40 at 12 months and 300 individuals in service at baseline, 352 at 6 months, and 347 at 12 months. Three subscales were derived: Clinical (e.g., establish goals, discuss medication instructions), Empathy (e.g., offer empathy, support individual-clinician communication), and Information (e.g., provide information, educate families on mental health). Confirmatory factor analysis yielded chi-square p-value = 0.09 indicating a good fit. Staff ratings for Empathy were significantly higher at 12 months v. baseline (p = 0.008) and on average were higher than individual report (p = 0.004). The same pattern was seen for Information (p = 0.007), but on average staff ratings were lower than reported by individuals (p < 0.0001). Clinical skills were not expected to change and did not. Discrepancies between staff and individual reports will be further investigated.

## Statement

Assessment of specific skills acquired during training can be monitored and compared to report from those receiving services to confirm adoption of a new practice and discover needed improvements.

## Funding

Community Care Behavioral Health Organization.

